# Congenital Cleft Earlobe: Classification and Surgical Techniques with a Scoping Review of the Literature

**DOI:** 10.3390/jcm15010359

**Published:** 2026-01-03

**Authors:** Young Joon Jun, Yeong Jun Park, Mi Ra Kim, Moo Jin Baek, Shin Hye Kim

**Affiliations:** 1 Department of Otolaryngology-Head and Neck Surgery, Uijeongbu Eulji Medical Center, Eulji University College of Medicine, Uijeongbu 11759, Republic of Korea; syrrjun@gmail.com; 2 Department of Otolaryngology-Head and Neck Surgery, Haeundae Paik Hospital, Inje University College of Medicine, Busan 48108, Republic of Korea; gtgtyyu@gmail.com (Y.J.P.); enthns@naver.com (M.R.K.); mjbaek@inje.ac.kr (M.J.B.)

**Keywords:** ear deformity, cleft earlobe, congenital, classification, corrective method

## Abstract

**Background/Objectives**: Congenital cleft earlobes are a rare deformity with variable degrees of severity. Because no widely accepted classification or corrective methods exist, we report a case of a 7-year-old boy who presented with a right defective-type cleft earlobe that was successfully reconstructed. We also elucidate the various types of congenital cleft earlobes and corrective methods through a scoping review of the literature. **Methods**: PubMed searches were performed using the terms ear deformity and cleft earlobe. We analyzed 14 articles to establish a classification system for congenital cleft earlobe and to identify appropriate corrective methods. **Results**: Our case was successfully reconstructed using a simple local flap technique that can be easily applied to defective-type cleft earlobes of moderate to severe degree. Based on this case and our systematic analysis, we have synthesized existing classification systems and surgical techniques for correcting congenital cleft earlobes according to subtype. **Conclusions**: We present a unified classification system for congenital cleft earlobes consisting of four subtypes based on external appearance: defective type, simple type, tag with or without hypoplasia, and triple lobe. Various reconstructive techniques—including simple suture, Z-plasty, Y-V advancement, and flap procedures—can be applied according to the specific earlobe subtype.

## 1. Introduction

The auricle is formed between the fifth and ninth weeks of gestation during the embryonic period. Growth of mesenchymal tissue from the first and second branchial arches produces six hillocks around the primitive meatus that fuse to form the auricle [1]. Congenital auricular deformities are relatively common and most frequently involve the upper one-third of the auricle; however, congenital earlobe deformities are rare, occurring in approximately 1 in 15,000 live births [2].

The cause of congenital earlobe deformities is not definitely known, and these deformities generally occur unilaterally. Congenital earlobe deformities present in a variety of forms, the most common being cleft earlobe, duplicate earlobe, absent earlobe, and earlobe with a skin tag. Abnormal fusion of hillock 1 (anterior portion of the earlobe) and hillock 6 (posterior portion of the earlobe) is probably the cause of cleft earlobe [3]. Absence of hillock 6 may lead to an absent earlobe in which a significant portion of the earlobe is missing.

To date, various surgical techniques to correct congenital cleft earlobe deformities have been reported. However, there are no standard guidelines for their application, and many of the corrective methods are not ideal because of problems such as conspicuous scars or incomplete correction of the cleft earlobe. In this article, we introduce a simple local flap technique to correct a defective type of congenital cleft earlobe. It can be easily applied in other cases with defective type of congenital cleft earlobe. Moreover, we organized various reconstructive methods through a scoping review of the literature.

## 2. Materials and Methods

### Search Criteria

This study reporting a case of a 7-year-old boy who presented with a right defective type cleft earlobe was approved by the Institutional Review Board of the Clinical Research Institute at our hospital (2022-08-027). Informed consent was obtained from the participant’s parent. To clarify the various types of congenital cleft earlobes and possible corrective methods reported to date, we adhered to the PRISMA (Preferred Reporting Items for Systematic Reviews and Meta-Analyses) guidelines when performing PubMed (http://www.ncbi.nlm.nih.gov/pubmed/, accessed on 25 August 2022) searches to identify all studies on cleft earlobe. The keywords used were ‘ear deformity’ and ‘cleft earlobe’, and the search was limited to articles in the English, Korean, and Japanese languages.

## 3. Results

### 3.1. Our Case

**Figure 1:**
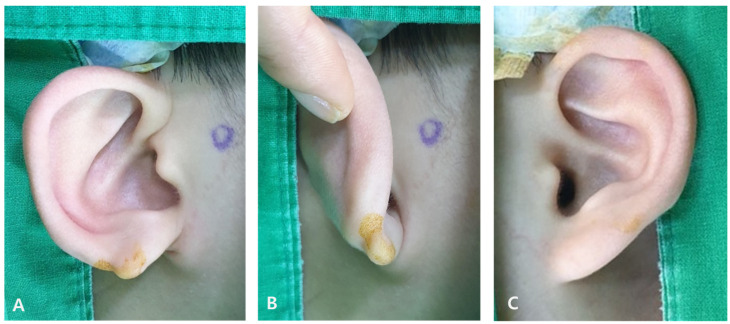
A 7-year-old boy with a defective type of congenital cleft earlobe on the right ear (**A**,**B**) and normal left ear (**C**).

A 7-year-old boy visited our outpatient clinic with a congenital cleft earlobe deformity, in which there was a small remnant of soft tissue in the infra-auricular area (Figure 1). The patient was otherwise healthy and denied any history of trauma to the ear or family history of congenital ear deformity. In comparison with the left side, there was considerable tissue defect in the right earlobe. The deformity was consistent with defective type cleft earlobe of moderate to severe degree as described by Kitayama and Park [4,5]. We planned to combine the small remnant soft tissue in the infra-auricular area with the defective earlobe using a local flap technique.

**Figure 2:**
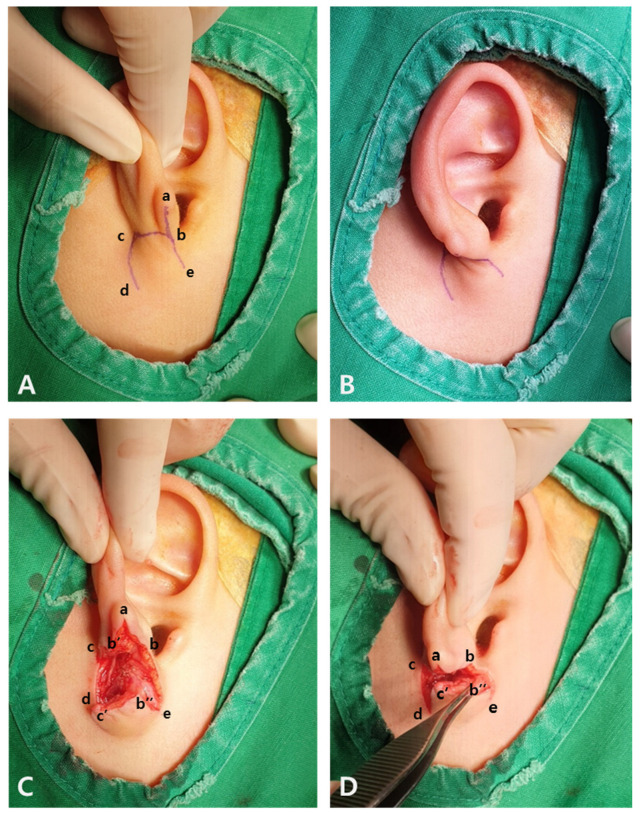
Surgical procedures using chair-shaped flap for infra-auricular tissue transposition.

The operation was performed under general anesthesia. The right auricle was compared with the left auricle, and the amount of tissue missing on the right side was determined. Markings for the skin incisions were made as shown in Figure 2A. A chair-shaped flap was designed to contain the infra-auricular remnant soft tissue, considering the size and volume of the defective earlobe. A full thickness incision was made, and points b′, b″, and c′ were generated. Point a was sutured to point c′ and point b′ was sutured to point d (Figure 2B–D). The lengths of all the designed lines were required to be equal to facilitate accurate alignment. The b–c length and c–d length were the same, but the a–b length was shorter than these. Therefore, an additional b–e incision was added, and point b″ was sutured slightly to the right side of point b′. The technique was a type of Z-plasty with b–e as an added incision.

**Figure 3:**
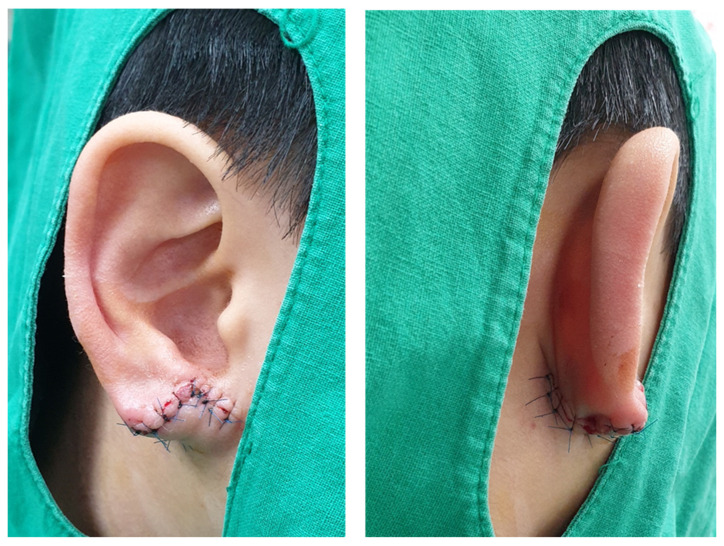
Postoperative appearance at 1 day.

**Figure 4:**
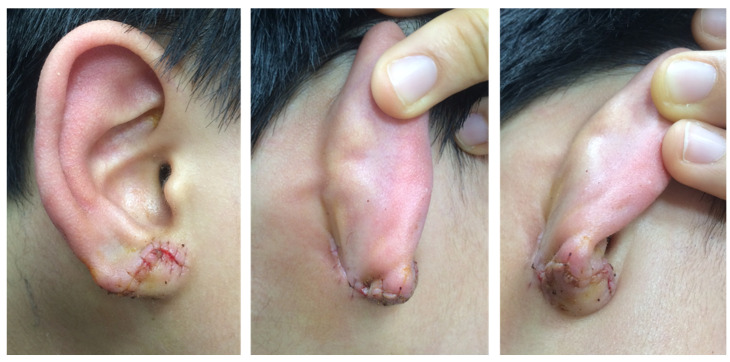
Postoperative appearance at 1 week (stitch-out state).

**Figure 5:**
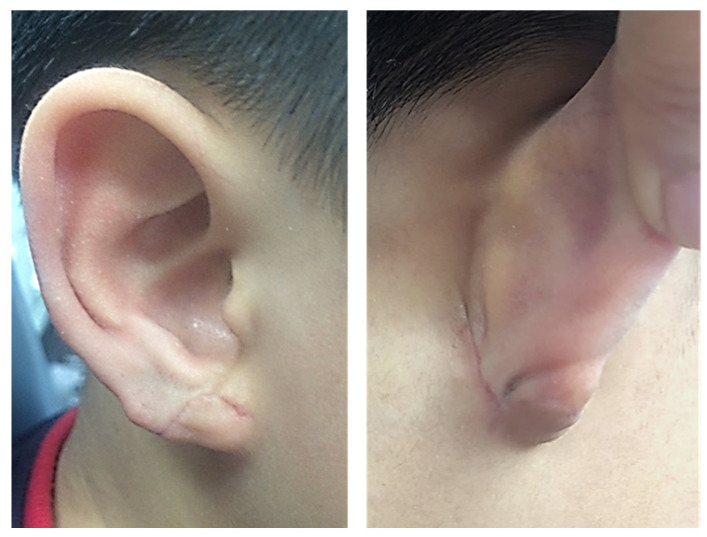
Postoperative appearance at 1 month.

**Figure 6:**
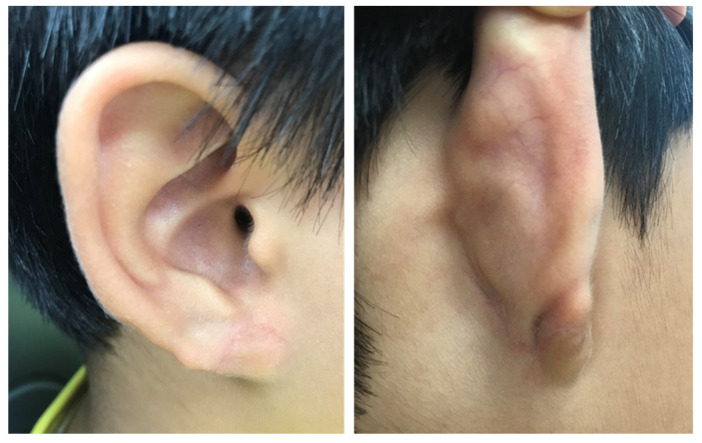
Postoperative appearance at 6 months.

The appearance of the earlobe on the first postoperative day is shown in Figure 3. The patient was evaluated at one week, one month, and six months postoperatively (Figure 4, Figure 5 and Figure 6). As compared with the contralateral side, the size and volume of the earlobe were similar and the appearance was symmetrical. The operative scar was acceptable, and other complications including scar contracture were not noted. The patient and his parents were very satisfied with the postoperative appearance. Longer follow-up is needed to assess the outcomes of scar maturation and contracture. Our technique can be easily applied in moderate to severe degrees of defective type cleft earlobe, since there is separated soft tissue which can be transposed to reconstruct a new earlobe.

### 3.2. Classifications and Surgical Techniques for Congenital Cleft Earlobe

Congenital cleft earlobe has been reported in many previous studies, and there are several classifications of congenital cleft earlobe. In 1980, Kitayama analyzed 30 cases of congenital cleft earlobe and classified them into 4 subtypes: longitudinal type (16 cases), triple-lobe (9 cases), transverse (4 cases), and defective (1 case) [4]. The triple-lobe type is a lobe divided into three lobules by a longitudinal and transverse cleft.

In 1999, Park analyzed 77 cases of lower auricular malformations, including 49 cases with cleft earlobe (63.6%), which was the most common deformity [5]. All 49 cases with cleft earlobe were affected unilaterally. Park classified them into four subtypes according to external appearance and the corrective methods required: defective type (34 cases), tag and cleft (5 cases), tag and cleft with hypoplasia (6 cases), and simple type (mild defective type, 4 cases). The longitudinal type classified by Kitayama corresponds to the defective type of mild degree in Park’s literature. The transverse cleft type classified by Kitayama includes ‘tag and cleft’ and ‘tag and cleft with hypoplasia’ subtypes in Park’s report.

To date, various reconstructive methods have been described according to the severity of the deformity, including several flap techniques, Y-V advancement, Z-plasty, and simple suture techniques. We organized previously reported reconstructive methods according to the four subtypes of congenital cleft earlobe deformity (Table 1).

#### 3.2.1. Defective Type (Moderate to Severe Degree)

For correction of defective type, a chondrocutaneous flap supplied by the middle division of the posterior auricular artery can be used [5]. Using this method, it is possible to maintain a good appearance of the earlobe in the long term with a less conspicuous donor scar.

Gavello used a postauricular bilobed flap and folded it to form the anterior and posterior aspects of the earlobe [6]. It is a simple, one-stage procedure, and suitable for earlobe defects of almost all sizes and volumes. The donor site can be repaired with primary closure and the scar at the postauricular mastoid region is not easily visible. However, flap contracture may develop as it is a long flap based on a relatively narrow pedicle.

Alanis used a vertical flap traced downward from the inferior end of the auricle to form the posterior aspect of the reconstructed earlobe [14]. This procedure is a simple, one-stage procedure, and the donor site can be primarily closed without a skin graft. However, the scar of the donor site and flap contracture may develop as with Gavello’s procedure since there is a long flap based on a relatively narrow pedicle.

#### 3.2.2. Longitudinal Type, Simple Type (Mild Defective Type)

Park suggested closure with Z-plasty to correct the simple type of cleft earlobe, even though it may result in a slight size difference between the normal and corrected earlobes [5]. Fujiwara reported successful reconstruction using a triangular flap repair technique in 16 patients with longitudinal clefts [7]. They derived the technique from the Tennison-Randall triangular flap operation for the cleft lip. No postoperative notching or contracture was observed in any of the 16 patients who underwent this procedure.

Maral used the Y-V advancement technique in vertical clefts [8]. The posterior lobe was transposed to form the free margin of the reconstructed earlobe, and the anterior lobe was divided into medial and lateral flaps to make the anteromedial and anterolateral aspects of the reconstructed earlobe. The main advantages of this technique are that the thickness and natural dimensions of the earlobe can be reconstructed with a smooth surface on the free margin of the earlobe without scar formation, and reconstruction can be done with minimal sacrifice of earlobe tissue. This technique was not recommended in cases with severe soft tissue deficiency.

Lee suggested a two-flap and Z-plasty technique for correction of longitudinal clefts [9]. Two rectangular flaps were designed along the margin of the cleft earlobe. In the lateral lobe, the posteriorly based de-epithelialized flap was elevated, while an anteriorly based de-epithelialized flap was created in the medial lobe. Each de-epithelialized flap was fixed into subcutaneous pockets, which were made on the anterior surface of the lateral lobe and the posterior surface of the medial lobe. A small Z-plasty was done at the earlobe margin.

Karaci reported a longitudinal incision and simple suture method for the longitudinal type of cleft earlobe [10]. The marking for the incision was drawn longitudinally from the top of the cleft to the bottom of the medial component. Lateral and medial margins were matched, and the two divided surfaces were sutured anteriorly and posteriorly. This method needs no additional incision outside the cleft margin, but it is not appropriate in the case of acquired split earlobe deformities due to the wide cleft surface.

#### 3.2.3. Transverse Type, Tag and Cleft with or Without Hypoplasia

Park suggested a buttoning procedure (button-down or button-up method) for tag and cleft [5]. In a case of tag and cleft with hypoplasia, the tag was buttoned down into a slit on the posterior cleft earlobe after removal of posterior skin. Then, a chondrocutaneous postauricular arterial flap was transposed to the defect to form a symmetric earlobe. The chondrocutaneous postauricular arterial flap requires more experience and skill for the surgical technique, and the donor site requires a skin graft for closure. Park suggests that using a skin flap with cartilage grafting is better than using a skin flap only, because the grafted cartilage prevents scar contracture of the transferred skin flap.

Qing reported the diametric hinge flaps method in 4 cases with longitudinal or transverse type [11]. One flap is based on the superior edge of the cleft and should be dissected from back to front, as if opening a book or hinge; the other flap is based on the inferior edge and should be dissected from front to back, as if opening a second diametrically opposed book or hinge. The superior hinge flap covers the defect produced by opening the inferior hinge flap, and the inferior hinge flap covers the defect produced by opening the superior hinge flap. Then, a small bolster external fixation is performed in the central part of both page-turning flaps.

#### 3.2.4. Triple-Lobe

The primary problems in triple-lobe type are the different anatomical planes and poor soft tissue connection of the three components. Basat presented a surgical method for triple-lobe type defects with anterior, posteromedial, and lateral earlobe components [12]. The three components were dissected, and the lateral component was transferred toward the anterior component. The posteromedial component was transferred medially to align with the lateral component, and all three components were sutured subcutaneously.

Stanley reported a more complicated case of triple-lobe type defect with four components [13]. An incision was made from the posterior to the transverse cleft creating bookend flaps, and the inferior lobe was de-epithelialized and sutured with the other lobes to make one earlobe with sufficient volume.

## 4. Discussion

As the size and the shape of normal auricles vary, the degree of deformity can also vary. For this reason, there appears to be no widely accepted classification for cleft earlobe. Our review can help clarify the various types of congenital earlobe deformities and possible corrective methods reported to date. In this review, we unified the classification for cleft earlobe by matching the subtypes for congenital cleft earlobe classified by Kitayama and with those classified by Park [4,5]. Congenital cleft earlobe can be subdivided into four categories by their configuration: (1) defective type (moderate to severe degree), (2) simple type (mild defective type), longitudinal type, (3) tag and cleft with/without hypoplasia, transverse type, and (4) triple lobe type (combination of longitudinal and transverse cleft).

There have been no established corrective methods for each subtype of cleft earlobe. In this review, we organized the corrective methods according to the four subtypes based on the reported literature. To correct congenital clefts, we have to appreciate that corrective methods for congenital clefts differ from those for acquired clefts in two aspects. First, congenital clefts show a wide range of severity and localization, from simple notching to cases with extensive tissue deficiencies, whereas most acquired clefts have almost no tissue deficiencies [10]. Second, congenital clefts often have components of different sizes on either side of the cleft, whereas most acquired clefts involve equal volume components [11]. The focus of corrective methods for congenital clefts is to make a geometric operation plan that can lead to a natural round lobar shape with an appropriate amount of tissue.

In our pediatric patient with moderate-to-severe defective type cleft earlobe, a kind of local flap technique (infra-auricular transposition flap), which called chair-shaped flap, was used. This technique offers several advantages: it can be performed in a single procedure, the donor site is primarily closed with the resulting scar well concealed along the postauricular crease, and it utilizes minimal soft tissue remnant without leaving a visible donor site scar. Furthermore, unlike Gavello’s procedure and Alanis’s procedure, this technique does not result in flap contracture [6,14].

To apply a uniform standard corrective method to each subtype of cleft earlobe can be paradoxical. The method of repair should be chosen according to the extent of the deficient tissue. There were commonly accepted principles throughout the reviewed literature that a surgical procedure should preserve the original volume of the soft tissue and should restore the natural curvature of the defective auricle compared to the contralateral side.

## Figures and Tables

**Table 1:** Corrective methods for 4 subtypes of congenital cleft earlobe.

Type	Configuration	Description	Corrective Methods
Defective type (of moderate to severe degree) [4,5]	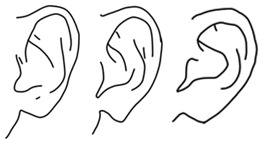	Earlobe seems to be defected with a wide cleft.	Chondrocutaneous postauricular arterial flap [5]
			Postauricular bilobed skin flap [6]
			Postauricular vertical flap [7]
			Chair-shaped flap with infra-auricular soft tissue (this article)
Longitudinal type [4]	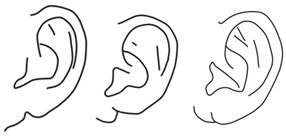	The cleft run from the free margin of the lobe to the intertragic notch.	Z-plasty [5]
			Triangular flap [7]
			Y-V advancement [8]
Simple type (defective type of mild degree) [5]			Two flaps and Z-plasty [9]
			Simple correction [10]
			Diametric hinge flap [11]
Transverse type [4]	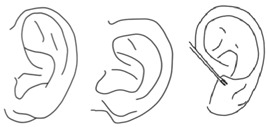	The cleft runs horizontally, and the lobe is divided the anterior and posterior lobe.	Buttoning procedure (for tag and cleft) [5]
Tag and cleft [5]			Chondrocutaneous postauricular arterial flap (for tag and cleft with hypoplasia) [5]
Tag and cleft with hypoplasia [5]			Diametric hinge flap [11]
Triple-lobe [4]	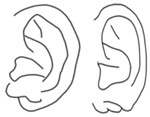	The lobe is divided into three lobules by a longitudinal and transverse cleft.	Dissection and transfer technique (three or four lobes together) [12,13]

## Data Availability

The original contributions presented in this study are included in the article. Further inquiries can be directed to the corresponding author(s).
